# Mental health in the workplace: The investor perspective

**DOI:** 10.1371/journal.pmen.0000156

**Published:** 2024-10-09

**Authors:** Amy Browne

**Affiliations:** CCLA Investment Management, London, United Kingdom; PLOS: Public Library of Science, UNITED KINGDOM OF GREAT BRITAIN AND NORTHERN IRELAND

In 2017 the then UK Prime Minister, Theresa May, commissioned an independent review into how employers could better support the mental health of all people in employment. The resulting Thriving at Work review revealed that the UK was facing a mental health challenge at work that was much larger than anyone had predicted [1].

Thanks to Deloitte, the report put hard and fast figures on the cost to employers of mental ill-health. The mist of sweeping generalisation was blown away to reveal an astonishing reality: that companies are losing billions of pounds because their employees are less productive, off sick, or leaving work altogether.

At the time, the cost of mental ill-health to UK businesses was estimated at £33bn-£42bn each year [1]. In 2020–21, as the Covid pandemic swept across the globe, that figure had risen to £55bn. By 2022–23 it had eased back to £51bn; relatively lower, but staggeringly high when considered in absolute terms [2]. The associated human cost is immeasurable.

Be that as it may, there are reasons to be positive. Research published in 2024 found that investing in employees’ mental health yields positive financial results. The average return on investment is estimated at £4.70 for every £1 spent; an amazing 370% return on invested capital [2]. This represents a phenomenal opportunity, not only for employers, but also for their many internal and external stakeholders: employees, customers, suppliers, creditors, communities, governments, and, not least, their investors.

## The CCLA Corporate Mental Health Benchmark

As part owners of the companies in which they buy shares, investors are in a unique position to influence corporate behaviour for the better. It is also the industry’s interests to be a force for good, since today’s sustainability challenges will become the economic headwinds of tomorrow.

Driven by clear evidence that improving the mental health of an organisation saves money, CCLA Investment Management set out to change the accepted way in which listed businesses approach the mental health of their people [3].

The CCLA Corporate Mental Health Benchmark, launched in 2022, aims to inform and accelerate progress in this area; an area that has historically been hidden behind closed doors in the workplace. Now in its third year, the benchmark evaluates more than 200 listed companies, with a combined 24 million employees, annually, and ranks them into five performance tiers [4]. Performance is assessed against a set of 27 assessment criteria developed by mapping existing frameworks and reference sources, and with the support of an independent expert advisory panel [5]. In 2023, an additional gap analysis exercise was undertaken to ensure that the criteria fully reflected the most up to date guidance from the World Health Organisation (WHO) and ILO [6–8].

Importantly, the benchmark does not attempt to gauge the ‘happiness level’ of a company’s workforce. Rather, to encourage employers to create the conditions under which workers can thrive. This necessitates a 360° view of mental health, from thriving, to struggling, to not coping. It also requires clear leadership commitments, watertight policies and cohesive workplace programmes that equip people with the knowledge and skills to support their own (and others’) mental health.

To raise the profile of the benchmark, and to incentivise businesses to implement the framework, CCLA has built a sizeable global investor coalition on workplace mental health. The coalition now comprises 55 investor signatories with a combined $9.9 trillion in assets under management [9].

We acknowledge that benchmarking is a long game and, at just three years old, this benchmark is at a formative stage. Nonetheless, it appears to be doing its job in driving corporate performance on workplace mental health at a systemic level. It has mobilised the investment community into action, it has incentivised many businesses to improve, and the ability to assess and compare companies on their approach enables investors to track corporate progress over time.

## Outcomes

The CCLA Corporate Mental Health Benchmark was first published in 2022, with two separate company rankings: a ‘UK 100’ [10] and a ‘Global 100’ [11]. In 2023, companies were assessed for the second time, allowing a year-on-year comparison.

In 2023, we assessed 211 listed companies, across the two benchmarks, on their approach to workplace mental health [12, 13]. One hundred and eighteen of them engaged directly with CCLA on this topic and 42 companies demonstrated improvement sufficient to result in a move up by one or more performance tiers, to the implied benefit of their ~7 million employees worldwide. Those 42 ‘improver’ companies are set out in Table 1 below.

**Table 1:** 

Amazon.com Inc
Ashtead Group PLC
Bristol-Myers Squibb Co
China Construction Bank Corp
CRH PLC
Diageo PLC
DS Smith PLC
Dunelm Group PLC
Entain PLC
Experian PLC
Glencore PLC
Hermès International
HSBC Holdings PLC
IMI PLC
Johnson Matthey PLC
JPMorgan Chase & Co
Kingfisher PLC
LVMH Moet Hennessy Louis Vuitton SE
Mastercard Inc
Meituan
Mondi PLC
Morgan Stanley
NatWest Group PLC
NIKE Inc
Novartis AG
Novo Nordisk A/S
Rentokil Initial PLC
Rio Tinto PLC
Roche Holding AG
Shell Plc
SSE PLC
SSP Group Plc
Thermo Fisher Scientific Inc
Toronto-Dominion Bank
TotalEnergies SE
Toyota Motor Corp
TUI AG
Unilever PLC
Walmart Inc
Weir Group PLC
Whitbread PLC
WPP PLC

## Recommendations for employers

We encourage all listed companies, regardless of size, location, or industry, to review the findings in the benchmark reports. Specifically, we recommend the following:

1. **Demonstrate a leadership commitment to mental health at the highest level of management.** CEOs can play a critical role in driving a culture of openness and transparency around mental health and efforts to destigmatise the subject could be amplified if a greater number of CEOs were to speak out.
2. **Publish a mental health at work policy and ensure that its scope is clear.** It is good practice for companies to formalise their approach to workplace mental health in a policy (or equivalent document). While the existence of a policy may not provide a guarantee of implementation or supportive practices, the absence of a policy is a clear sign that workplace mental health is not firmly on the business agenda.
3. **Set mental health-related objectives or targets and report on progress against them.** Objectives and targets are the point where policy commitments are translated into action, and where resources and responsibilities are allocated for their delivery. Investors place significant value on published objectives as a sign of a company’s commitment to continuous improvement. Investors also recognise that published objectives provide an important accountability mechanism as they create an expectation that companies will report on progress against their stated commitments.
4. **Provide employees with good working conditions.** Good working conditions can help to prevent new mental health problems arising and support people with existing conditions to get on in work and thrive. CCLA’s benchmark methodology is mapped against international standards and management frameworks for workplace mental health, recognising six key ‘good work’ principles: diversity, equity and inclusion; fair pay and financial wellbeing; board-employee information and consultation; flexible working; career progression and job adjustment; and anti-bullying and non-harassment.
5. **Promote mental health awareness among employees and involve them in the design and delivery of mental health programmes.** In addition to having a clear policy on mental health in the workplace, companies should develop and deliver systemic programmes of activity that raise awareness and promote an understanding of mental health in the workplace. Enabling employees to contribute to the design and development of effective mental health initiatives helps to facilitate an open and progressive culture for mental health.
6. **Equip managers with the necessary skills and training to support employee mental health**. Mental health training for workers and managers is a key intervention recommended in the 2022 ‘WHO guidelines on mental health at work’. The effective implementation of a workplace mental health strategy relies on competent managers who are trained in mental health awareness and equipped to listen, reassure and respond appropriately to workers experiencing mental ill-health. These individuals are ideally placed to direct individuals to relevant support resources that might prevent progression to long-term sickness.
7. **Collaborate with others through industry and/or academic partnerships to promote positive workplace mental health.** Workplace mental health is a collective matter for businesses and their industry sectors, as well as being an individual concern for companies to manage. Making progress and raising standards internationally requires individual companies to support academic research and other development programmes to improve workplace mental health; share knowledge and expertise with industry peers; support public policy debates around workplace mental health; and support industry and stakeholder initiatives directed at improving workplace mental health.

This is our message to employers: promoting and investing in the mental health of your workers is both a moral and an economic imperative. By directing resources thoughtfully, not only will you improve the quality of your people’s lives, but you will also build a workforce that is more productive, more resilient and more profitable.
